# P-1502. Barriers and Enablers for Tdap Vaccination Among Pregnant Women Attending Antenatal Clinics in a Tertiary Hospital

**DOI:** 10.1093/ofid/ofaf695.1686

**Published:** 2026-01-11

**Authors:** Jomel Raju, Leena James, Maria Tom

**Affiliations:** St. Joseph's College of Pharmacy, Cherthala, Pala, Kerala, India; St. Joseph's College of Pharmacy, Elampally, Kerala, India; St. Joseph's College of Pharmacy, Cherthala, Pala, Kerala, India

## Abstract

**Background:**

Vaccination with tetanus, diphtheria, and acellular pertussis (Tdap) during pregnancy is a proven strategy to prevent neonatal pertussis-related morbidity and mortality. However, in India, Tdap vaccination uptake during pregnancy remains low, with limited research on the behavioral and systemic barriers. This study aimed to evaluate the barriers and facilitators influencing Tdap vaccination among pregnant women attending antenatal care (ANC) in a tertiary care hospital in South India.

**Figure:**
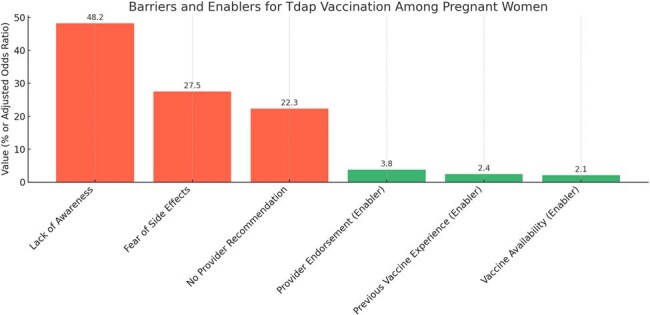
Barriers and Enablers for Tdap Vaccination Among Pregnant Women This chart visualizes the key barriers (in red) and enablers (in green) influencing Tdap vaccination uptake among pregnant women in a tertiary care hospital in South India. Barriers include lack of awareness (48.2%), fear of side effects (27.5%), and absence of provider recommendation (22.3%). Enablers such as provider endorsement (aOR: 3.8), previous positive vaccine experience (aOR: 2.4), and vaccine availability in the clinic (aOR: 2.1) significantly supported uptake.

**Methods:**

A cross-sectional mixed-methods study was conducted between January and August 2024 at the ANC unit of a 650-bed tertiary care teaching hospital in rural Kerala. Quantitative data were collected using a structured questionnaire administered to 382 pregnant women in their second or third trimester. Multivariate logistic regression was used to identify independent predictors of vaccination. In-depth interviews (n=20) were conducted with participants and healthcare providers to explore contextual factors. Statistical significance was set at p < 0.05.

**Results:**

Overall, only 36.9% of eligible women had received Tdap vaccination. Key barriers included lack of awareness (48.2%), fear of side effects (27.5%), and absence of provider recommendation (22.3%). Enablers included strong provider endorsement (aOR: 3.8, 95% CI: 2.2–6.5), previous positive vaccine experience (aOR: 2.4, 95% CI: 1.5–4.0), and availability of vaccine at ANC clinic (aOR: 2.1, 95% CI: 1.3–3.4).

**Conclusion:**

Improving Tdap coverage among pregnant women requires targeted education, proactive provider recommendation, and logistical support for vaccine availability. Antenatal clinics in resource-limited hospitals should integrate Tdap education as a standard of care.

**Disclosures:**

All Authors: No reported disclosures

